# Psychoeducation for Caregivers of Individuals With Borderline Personality Disorder: A Randomized Controlled Trial of Multiple Family Group Therapy

**DOI:** 10.1002/pmh.70029

**Published:** 2025-07-01

**Authors:** Silvia Tempia Valenta, Biancamaria Bortolotti, Francesca Martino, Paola Tedesco, Anna Rita Atti, Domenico Berardi, Diana De Ronchi, Marco Menchetti

**Affiliations:** ^1^ Department of Biomedical and Neuromotor Sciences University of Bologna Bologna Italy; ^2^ Research Group on Emergency and Disaster Medicine Vrije Universiteit Brussel Brussels Belgium; ^3^ Department of Mental Health and Pathological Addictions Bologna Local Health Unit Bologna Italy

**Keywords:** borderline personality disorder, caregiver, communication skills, family, group therapy, mental health, psychoeducation, RCT, social functioning

## Abstract

Multiple family group (MFG) therapy is a psychoeducational intervention designed for caregivers of individuals with borderline personality disorder (BPD). This study is the first to compare MFG therapy with treatment as usual (TAU). The objectives were to assess MFG therapy's impact on caregivers' social functioning, perceived support from mental health professionals, coping and communication skills development, and overall satisfaction with the program. A randomized controlled trial (RCT) was conducted at the West Community Mental Health Center in Bologna, Italy. This study enrolled 57 caregivers of 48 adult patients diagnosed with BPD. Participants were randomly assigned to the MFG or TAU intervention. Psychometric assessments were conducted at baseline (T0) and 6 months (T1). Between‐group differences in improvements were analyzed using independent sample *t*‐tests and linear mixed model (LMM) analyses to account for unequal group sizes and missing data. Compared to the TAU group, caregivers completing the MFG program reported significant improvements at T1 in social functioning, perceived support from mental health professionals, and family coping skills, including improved access to information and a marked reduction in avoidance behaviors. LMM analysis showed that MFG participants improved more over time in social support, communication, and service satisfaction than controls. This study highlights that MFG therapy has strong potential for improving social functioning and coping skills among caregivers of individuals with BPD. These findings support the feasibility and clinical relevance of integrating MFG programs into generalist mental health services.

## 1 Introduction

*Mental illness is a family affair* (Falloon 1987), a reality that applies across the spectrum of mental health conditions, from psychosis to personality disorders. Families supporting a loved one with a mental health condition often face a range of emotional, social, and practical challenges (Fadden et al. 1987). This is commonly described as “family burden,” defined as “all the difficulties experienced by families as a consequence of someone's illness” (Sales 2003). The impact of this is felt throughout family life, affecting everything from daily routines and leisure activities to financial stability, health, and relationships (Baronet 1999).

Caregivers of individuals with borderline personality disorder (BPD) may perceive particularly high levels of burden, largely because of the disorder's intense interpersonal difficulties (Scheirs and Bok 2007; Bailey and Grenyer 2013; Bailey and Grenyer 2014; Seigerman et al. 2020). Recent literature continues to highlight the significant psychological and somatic distress experienced by these caregivers, including grief, sadness, guilt, and powerlessness (Jørgensen et al. 2021; Meshkinyazd et al. 2021). Emerging studies have begun to explore mechanisms underlying this burden. For example, Fonseca‐Baeza et al. (2023) demonstrated that difficulties in emotion regulation may mediate the relationship between caregiver burden and negative mental health outcomes, such as depression and anxiety (Fonseca‐Baeza et al. 2023). Additionally, Hayes et al. (2024) found that caregivers who assumed leadership roles in support interventions reported improved well‐being and reduced psychological distress compared to non‐leader participants (Hayes et al. 2024).

BPD is a personality disorder characterized by an ongoing pattern of impulsive behaviors and great instability regarding emotions, self‐image, and interpersonal relationships (American Psychiatric Association 2013). These clinical features often entail intense anger, self‐harm, and suicidal behavior along with relationship issues with relatives and significant others (American Psychiatric Association 2013; Gunderson et al. 2018). Recent literature estimates that BPD affects approximately 0.7%–2.7% of the general adult population, with higher rates observed in clinical settings, around 12% in outpatient and up to 22% in inpatient psychiatric services (Leichsenring et al. 2024). Although the general prognosis, contrary to what was thought in the past, is in the long term more positive than expected (Zanarini et al. 2012), BPD remains a major individual and public health concern, as it is associated with considerable functional impairment (Fruzzetti et al. 2005; Terzi et al. 2017; Bohus et al. 2021), poor physical health status (Douzenis et al. 2012; Shen et al. 2017), and difficulties engaging in treatment (Chiesa et al. 2010; Martino et al. 2012).

Family members are often greatly affected by the consequences of BPD and at the same time play a role in influencing the well‐being of the person suffering from this disorder (Hoffman et al. 2007; Bailey and Grenyer 2013). Despite the high prevalence of BPD, quantitative research on family burden in this context remains limited, especially when compared to the more extensive literature on other mental health conditions (Bailey and Grenyer 2013; Kirtley et al. 2019). The limited number of existing studies demonstrates that the family burden on caregivers of BPD is directly attributable to impulsive behaviors and emotional instability because these traits often result in acute conflicts, poor communication, and labile emotional attachment (Gunderson and Lyoo 1997; Hoffman et al. 2003; Ekdahl et al. 2011; Martino et al. 2014). High levels of stress can also arise in caregivers in response to their loved one's experiences with self‐harming or suicidal behaviors. These situations often contribute to chronic stress, anxiety, emotional exhaustion, and a state of hypervigilance, as caregivers attempt to provide support while managing ongoing uncertainty and emotional strain (Giffin 2008; Ekdahl et al. 2011).

While therapeutic interventions for individuals with BPD have evolved significantly over the years, family‐focused treatments remain limited. In contrast to other psychiatric disorders, where structured support services for both patients and their families are more commonly available, families of individuals with BPD are often underserved and overlooked (Hoffman et al. 2003; Sansone and Sansone 2009). Since the early 2000s, a small number of standardized interventions specifically designed to reduce the impact of BPD on relatives have been formally evaluated (Hoffman et al. 2007; Guillén 2024). Multiple family group (MFG) is a psychoeducational treatment designed to support families in coping with the challenges of having a family member with mental health difficulties, within a non‐judgmental environment (Falloon et al. 1981; Lefley 1987). The program builds on significant therapeutic innovations, including Lefley's (1987) informal psychoeducation and McFarlane's multifamily group structure designed for families in need (Lefley 1987; Dixon et al. 2001; McFarlane et al. 2003). Gunderson et al. (1997) introduced the MFG model for BPD within a psychoeducational framework that focused on informing families about the disorder, teaching behavioral strategies for managing its challenges, improving family communication, reducing family conflict, and supporting coping strategies (Gunderson et al. 1997). The MFG intervention was originally developed and piloted at McLean Hospital, Harvard Medical School, in 1997 by Gunderson and colleagues, specifically for families of individuals with BPD (Gunderson et al. 1997). Although the pilot study suggested promising outcomes, these preliminary findings have not been systematically replicated or evaluated in other settings (Gunderson et al. 1997; Hofmann and Tompson 2004).

To our knowledge, this study is the first to evaluate the impact of MFG therapy for caregivers of individuals with BPD after the pilot study (Gunderson et al. 1997). The primary objectives were to examine differences between MFG therapy and treatment as usual (TAU) in terms of (1) caregivers' social functioning and (2) perceived support from mental health professionals. Secondary objectives included exploring (3) coping and communication skills among caregivers and their (4) satisfaction with the intervention. We hypothesized that social functioning and perceived support from mental health professionals would have been higher in the MFG group compared to the TAU group. Additionally, it was hypothesized that caregivers in the MFG group would have reported better coping and communication skills, as well as higher satisfaction with the intervention, compared to those in the TAU group.

## 2 Methods

### 2.1 Study Design and Participants

This randomized controlled trial (RCT) was conducted between 2012 and 2017 in three community mental health centers (CMHCs) in the western part of Bologna, within the local Department of Mental Health and Pathological Addictions. CMHCs provide evaluation, outpatient treatments, and, if necessary, referral to semi‐residential/residential centers, and inpatient services for a broad spectrum of mental disorders. The Ethical Committee of Bologna Local Health Unit study approved the protocol on April 22, 2011 (Ethical Committee code = 11,015).

This study enrolled (1) adult patients (over 18 years old) with a clinical diagnosis of BPD according to the DSM‐5 criteria who were willing to participate in the research project and had accepted to involve a caregiver and (2) caregivers likewise willing to participate in this study. Exclusion criteria were poor understanding of Italian language, ongoing psychotic disorders, cognitive impairment or intellective disability, or acute clinical situations that prevented explaining the protocol or obtaining informed consent.

Both patients and their caregivers were asked to provide informed consent to participate in this study. While dual consent was preferred, cases in which only the caregiver provided consent were still included, as this study aimed to assess caregivers' psychometrics independently of patient participation. Although this situation did not occur in practice, this study design allowed for the inclusion of caregivers in such cases.

### 2.2 Measures

At baseline (T0), patients and caregivers filled out sociodemographic and clinical forms. Patients were also administered Structured Clinical Interview for DSM‐IV Axis II Personality Disorders (SCID‐II) (Glasofer et al. 2017) to confirm a clinical diagnosis of BPD. Subsequently, recruited patients and caregivers received a psychometric evaluation through validated questionnaires. Assessment visits were scheduled at 6 months (T1, end of treatment). The assessment was conducted by interviewers not involved in the patient's treatment and trained to the use of instruments and scales.

#### 2.2.1 Caregivers' Assessment

The primary outcomes for caregivers were assessed using the Social Network Questionnaire (SNQ) (Magliano et al. 1998) and the Family Problems Questionnaire (FPQ) (Magliano et al. 1998).

The SNQ, originally developed and validated in Italian by Magliano et al. (1998), is a 15‐item questionnaire evaluating the functionality of the social networks available to family caregivers (Magliano et al. 1998). The SNQ assesses key aspects of a caregiver's social environment, including the frequency of social interactions, availability of practical assistance, emotional and psychological support, and help from close relationships such as spouses or partners. The total score reflects the overall quality and strength of the caregiver's social network, with higher scores indicating greater support. The tool does not have standardized cut‐off values.

The FPQ, originally developed and validated in Italian by Magliano et al. (1998), investigates the experiences of family members in caring for a relative with a psychiatric disorder, measuring several aspects of the family burden (Magliano et al. 1998). The questionnaire assesses multiple aspects of caregiving, including perceived help received, emotional and psychological stress (subjective burden), and the practical impact of caregiving (objective burden), such as disruptions to daily life and financial strain. It also evaluates attitudes toward the patient, distinguishing between positive (empathic and supportive) and critical (negative or overprotective) views. The tool does not have widely established cut‐off scores.

Secondary outcomes for caregivers included improvements in coping and communication skills, measured by the Family Coping Questionnaire (FCQ) and the Italian ABC Questionnaire. Caregivers' satisfaction with the intervention was measured using the General Satisfaction Questionnaire (GSQ‐20.2).

The FCQ, originally developed and validated in Italian by Magliano in 1996, is a 34‐item questionnaire examining family members' coping strategies through 34 items grouped into subscales that measure various dimensions of coping (Magliano et al. 1996). The questionnaire explores coping strategies used by family members, including seeking information about the patient's condition, exerting efforts to improve communication, and maintaining social interests outside of caregiving. It also assesses more challenging dynamics, such as the use of coercion or tendencies to avoid conflict with the patient. Higher scores in each area reflect greater reliance on that particular coping strategy. The tool does not have standardized cut‐off values.

The ABC Questionnaire, originally developed and validated in Italian by Veltro in 2007, is a 23‐item scale that evaluates the support perceived by family members from health‐care services, their unmet needs, and the burden associated with caregiving for a relative with severe mental illness (Veltro et al. 2007). The total score provides an overall evaluation of the support received, unmet needs, and perceived burden. Higher satisfaction scores reflect positive perceptions of the services provided. There are no universal cut‐off values.

The GSQ‐20.2, developed by Huxley in 1991, is a 20‐item self‐report tool designed to evaluate overall satisfaction with health‐care services and the quality of treatment received (Huxley and Mohamad 1991). Higher scores indicate greater satisfaction with the intervention. There are no standardized cut‐off values. GSQ‐20.2, although widely used internationally, lacks formal Italian validation studies.

#### 2.2.2 Patients' Assessment

Patients were assessed using the Zanarini Rating Scale for BPD (ZAN‐BPD), the Difficulties in Emotion Regulation Scale (DERS), the Aggression Questionnaire (AQ), and the Self‐Harm Inventory (SHI).

The ZAN‐BPD is a nine‐item tool developed by Zanarini et al. (2003) to assess BPD symptom severity across four domains: affective, cognitive, impulsivity, and interpersonal functioning (Zanarini et al. 2003). Scores range from 0 to 36, with 8 or higher indicating clinically significant symptoms (Zanarini et al. 2003). Despite its international use, the ZAN‐BPD has not been formally validated in Italian populations.

The DERS, developed by Gratz and Roemer (2004), is a 36‐item self‐report tool that measures individuals' ability to regulate intense negative emotions. The Italian version, validated by Sighinolfi et al. (2010), assesses emotional regulation, control, and expression (Gratz and Roemer 2004; Sighinolfi et al. 2010). A total score above 80 suggests clinically significant difficulties in emotion regulation.

The AQ, by Buss and Perry (1992), is a 29‐item self‐report tool measuring tendencies toward aggression, anger, and hostility, validated in Italian by Fossati et al. (2003) (Buss and Perry 1992; Fossati et al. 2003). There are no universal cut‐off values, but higher total scores indicate greater aggression levels.

The SHI, developed by Sansone et al. (1998) and validated in Italian by Cerutti et al. (2012), is a 22‐item yes/no self‐report questionnaire assessing the presence and severity of self‐injurious behaviors. Its clinical use includes a cut‐off score of 5, which predicts a strong likelihood of self‐harm or self‐injurious behaviors (Sansone et al. 1998; Cerutti et al. 2012).

### 2.3 Block Randomization

A centralized randomization procedure was carried out by a researcher who was independent of the CMHC staff. Participants were randomly assigned to either the experimental group (MFG) or the control group (TAU) using a block randomization method. This approach, while resulting in unequal group sizes, was chosen to ensure a manageable distribution of participants across treatment cycles, reflecting the practical constraints of real‐world clinical settings. Specifically, block randomization was conducted in blocks of eight patients at a time (e.g., the first eight included, followed by eight not included, and so on), with the inclusion based on completed baseline assessments. Patients with incomplete or missing baseline assessments were excluded after the randomization process. CMHC clinicians contacted the external researcher to receive group allocation. Members of the control group were placed on a waiting list to receive MFG after the experimental group completed the program; following each treatment cycle, control participants entered the MFG intervention between 6 and 12 months after entering the waiting list.

### 2.4 Intervention

During this study, patients in both groups were made aware of the clinical assessment's results, and a personalized treatment plan was formulated including general psychiatric management, drug treatment, if necessary, individual psychotherapy in some cases, or rehabilitative interventions.

The MFG intervention consisted of a psychoeducational group composed of several families (from a minimum of three families to a maximum of eight) with 12 meetings, each lasting 1.5 h, and held on average every 2 weeks over a total duration of 6 months (Gunderson et al. 1997; Hofmann and Tompson 2004). The health‐care team consisted of a leader (a psychiatrist trained in MFG) and a co‐leader (a professional nurse also trained in MFG); the same two clinicians facilitated all MFG sessions consistently over the 5‐year period. The program required two initial training days conducted by an instructor experienced in the MFG technique, followed by two additional days of supervision for the facilitators, which occurred within the first 3 months of implementation to support the initial delivery of the intervention.

The program comprised three main phases. The first phase consisted of intensive family training sessions, held more frequently and with a structured format. These sessions focused on understanding the diagnosis of BPD and applying the family guidelines developed by Gunderson (Gunderson and Berkowitz 2006). In contrast, the second phase emphasized skill building and helped families develop practical strategies for managing challenges such as communication difficulties, anger regulation, and the patient's self‐injurious behaviors. The third and final phase was dedicated to consolidating changes, during which the therapist adopted a less directive role and encouraged participants to generalize the newly acquired skills through role‐playing exercises. Completion of the MFG program was defined as attending at least 70% of the scheduled sessions. Dropout was defined as attending less than 70% of the scheduled MFG sessions. Participants who met this criterion were considered dropouts and were not included in the final assessment, as follow‐up data were not collected from them. For details, please refer to Appendix I.

It is important to note that the duration and structure of the intervention that we employed were adapted from the original model developed by Gunderson, in order to better suit the needs of the participating families and the organizational context of the CMHCs. In Gunderson's original MFG program for BPD (1997), the intervention lasted for 1 year, and the authors did not specify the exact number of sessions (Gunderson et al. 1997). For organizational reasons, we structured our program to last 6 months with a total of 12 sessions. Furthermore, in our adaptation, patients were not included in the meetings, whereas Gunderson's MFG model involved inviting patients to participate in certain sessions.

In the TAU group, caregivers could be contacted by the referring psychiatrist, if needed, for brief family interventions or support. They were also placed on a waiting list to receive the MFG intervention after the experimental group completed the program. During the intervention period, families in the TAU group were monitored through regular follow‐up appointments with the clinical team. Dropout was defined as attending less than 70% of these scheduled follow‐up visits. As with the MFG group, participants classified as dropouts were not included in the final assessment, as no follow‐up data were collected from them.

### 2.5 Statistical Analysis

Data were analyzed using SPSS (Statistical Package for the Social Sciences) Version 24 for Windows. All outcome measures were quantitative and expressed as means with standard deviations (± SD). The level of statistical significance was set at *p* < 0.05. Descriptive statistics were calculated for caregivers and patients, including sociodemographic characteristics and baseline scores on outcome measures. An independent sample *t*‐test (two‐tailed) was used to compare the mean differences between the experimental (MFG) and control (TAU) groups at baseline (T0) and 6 months (T1).

To achieve 80% power with a significance level of 0.05, we estimated that 40 caregivers per treatment arm would be needed. However, for detecting an effect size of Cohen's *d* > 0.9, which reflects a very large effect, the required sample size for each group is approximately 18 participants. Because of practical constraints, our final sample size was smaller than initially planned, which may have impacted the study's power to detect the hypothesized difference. In light of this limitation, we decided to consider a Cohen's *d* of greater than 0.9 as the threshold for determining a meaningful effect size allowing for the detection of potentially clinically significant outcomes despite the reduced sample size.

Finally, to assess changes over time and evaluate the differential effects of the intervention, a linear mixed model analysis was conducted with group (MFG vs. TAU), time (T0 vs. T1), and their interaction as fixed effects, and a random intercept for each participant. This approach accounts for within‐subject correlations and missing data, allowing the inclusion of all available data, including partial cases, while accommodating individual variability and dropout.

## 3 Results

### 3.1 Participant Flow

**FIGURE 1:**
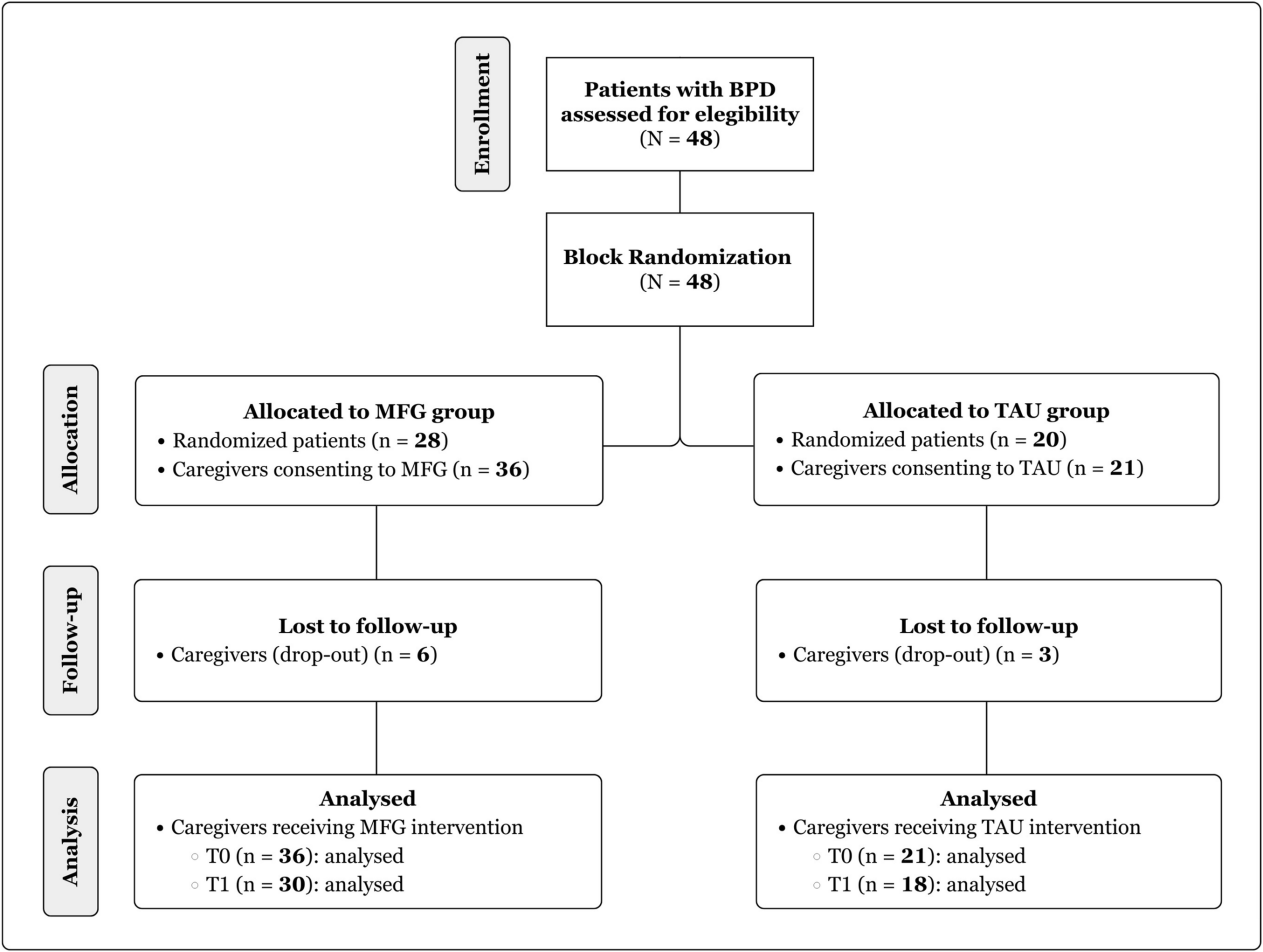
CONSORT flow chart. BPD, borderline personality disorder; MFG, multiple family group; TAU, treatment as usual.

A total of 48 BPD patients were randomly assigned to two groups through block randomization (Figure 1). The final distribution of patients into 28 and 20 per group, respectively, was due in part to the use of block randomization in groups of eight patients at a time. Additionally, some patients were excluded after randomization because of incomplete or missing baseline data, further contributing to the unequal group sizes.

Of the 28 patients in the experimental group, 36 corresponding caregivers participated in the MFG intervention. During the course of this study, six caregivers dropped out, resulting in 30 completing the post‐intervention assessment. In the control group, associated with the 20 patients receiving TAU, 21 caregivers were initially recruited. During the intervention phase, four caregivers dropped out, reducing the number who completed the post‐intervention assessment to 18.

### 3.2 Sociodemographic Characteristics

Participant demographics are detailed in Table 1. The mean age of family members (*N* = 57) was 52.6 ± 12.8 years, and they were primarily women (56.1%), with the majority being married (56.1%) and living with a partner or others (89.5%). Most family members had completed high school (40.4%), and a considerable percentage was employed full time (50.9%), reflecting a predominantly working‐age, educated sample with significant caregiving responsibilities. The majority of the family members participating in this study were mothers, accounting for 45.6% (*n* = 26) of the sample, followed by fathers at 22.8% (*n* = 13), and partners at 21.1% (*n* = 12). Siblings represented 7% (*n* = 4) of the participants, while other relatives made up 3.6% (*n* = 2).

**TABLE 1:** Baseline (T0) sociodemographic and psychometric profiles of caregivers.

Sociodemographics		MFG caregivers (*N* = 36)	TAU caregivers (*N* = 21)	*p* (chi‐square)
		*n*, *%*	*n*, *%*	
Gender	Female	21, 58.3%	11, 52.4%	0.662
	Male	15, 41.7%	10, 47.6%	
Education	Elementary school diploma	4, 11.1%	3, 14.3%	0.582
	Middle school diploma	10, 27.8%	6, 28.6%	
	High school diploma	15, 41.7%	8, 38.1%	
	University degree	7, 19.4%	4, 19.0%	
De facto civil status	Single	3, 8.3%	2, 9.5%	0.812
	Cohabiting	5, 13.9%	2, 9.5%	
	Married	19, 52.8%	13, 61.9%	
	Separated or divorced	4, 11.1%	3, 14.3%	
	Widowed	5, 13.9%	1, 4.8%	
Living situation	Living with partner or others	33, 91.7%	18, 85.7%	0.409
	Living alone	2, 5.6%	3, 14.3%	
	Living with the family of origin	1, 2.8%	0, 0.0%	
Occupation	Full‐time employment	16, 44.4%	13, 61.9%	0.242
	Part‐time employment	8, 22.2%	1, 4.8%	
	Employed in a supported position	1, 2.8%	1, 4.8%	
	Homemaker	3, 8.3%	4, 19.0%	
	Unemployed	1, 2.8%	0, 0.0%	
	Student	0, 0.0%	1, 4.8%	
	Retiree	7, 19.4%	1, 4.8%	

Psychometrics	Subscale	Mean *±* SD	Mean *±* SD	*p*‐value (Student's *t*‐test)
SNQ	Mean score	2.7 ± 0.5	2.5 ± 0.4	0.130
	Social contact frequency	2.5 ± 0.6	2.1 ± 0.6	0.027*
	Practical support	3.0 ± 0.8	2.7 ± 0.7	0.098
	Psychological support	2.8 ± 0.7	2.7 ± 0.9	0.502
	Support from close individuals	3.2 ± 1.2	3.4 ± 1.1	0.543
FPQ	Help received	2.5 ± 0.6	2.5 ± 0.4	0.954
	Objective burden	1.9 ± 0.6	1.7 ± 0.7	0.312
	Subjective burden	2.2 ± 0.7	2.0 ± 0.6	0.267
	Positive attitudes	2.7 ± 0.6	2.6 ± 0.9	0.738
	Critical or overprotective attitudes	2.0 ± 0.6	1.7 ± 0.7	0.032*
FCQ	Information	3.0 ± 0.9	2.3 ± 0.9	0.009**
	Communication	3.3 ± 0.6	3.6 ± 0.3	0.084
	Social interests	2.8 ± 0.7	2.5 ± 0.7	0.231
	Coercion	2.2 ± 0.7	1.6 ± 0.7	0.001***
	Avoidance	1.8 ± 0.8	1.3 ± 0.4	0.008**
ABC	Total score	86.0 ± 14.0	76.5 ± 15.8	0.025*
GSQ	Total score	36.2 ± 9.3	39.2 ± 10.5	0.334

Abbreviations: ABC, Italian ABC Questionnaire; FCQ, Family Coping Questionnaire; FPQ, Family Problems Questionnaire; GSQ, General Satisfaction Questionnaire 20.2; SNQ, Social Network Questionnaire.

Bold values marked with *, **, and *** indicate significant comparisons, which are denoted by 0.05, 0.01, and 0.001, respectively.

The patient group (*N* = 48) had a mean age of 29.5 ± 9.1 years and was composed mostly of women (79.1%), with a high proportion identifying as single (67.4%). Living arrangements for patients varied, although nearly half (46.5%) lived with their family of origin, and 39.5% lived with either a partner or roommates. Educational attainment among patients was also largely at the high school level (55.8%), but employment status differed significantly from the family group, with a large portion of patients being unemployed (34.9%) or, to a lesser extent, students (20.9%).

### 3.3 Patients' Psychometric Scores

Patients exhibited a mean ZAN‐BPD total score of 15.7 (± 5.0), reflecting moderate severity of BPD symptoms. Among its subscales, affective dysregulation scored 6.9 (± 2.3), cognitive dysregulation 3.2 (± 4.1), impulsivity 2.9 (± 2.3), and interpersonal relationships 3.9 (± 2.3). Emotional dysregulation, as assessed by the DERS, showed clinically significant levels with a total score of 107.8 (± 20.3). The AQ indicated moderate levels of aggression, with a total score of 85.8 (± 20.6). Patients also reported engaging in self‐harming behaviors, as reflected in the SHI total score of 8.4 (± 3.5).

### 3.4 Caregivers' Baseline Scores on Psychometric Outcome Measures (T0)

Participants' baseline scores on outcome measures are detailed in Table 1. Caregivers had a mean SNQ score of 2.6 (± 0.5), with social contact frequency (2.3 ± 0.6) and practical support (2.9 ± 0.8) showing moderate levels. Psychological support from the social network was 2.8 (± 0.8), and support from close individuals was 3.3 (± 1.2). The FPQ indicated that caregivers reported moderate levels of objective burden (1.9 ± 0.7) and subjective burden (2.1 ± 0.7). They also reported help received at 2.5 (± 0.6), positive attitudes at 2.6 (± 0.7), and critical or overprotective attitudes at 1.9 (± 0.6). The FCQ showed moderate scores in information (2.7 ± 1.0), communication (3.4 ± 0.5), and social interests (2.7 ± 0.7). Coercion was scored at 2.0 (± 0.7), and avoidance was at 1.6 (± 0.7). The ABC total score was 82.4 (± 15.3), and the GSQ total score was 35.8 (± 12.2).

### 3.5 Caregivers' 6‐Month Scores on Psychometric Outcome Measures (T1)

Participants' 6‐month scores on outcome measures are detailed in Table 2. Caregivers at T1 (*n* = 48; MFG *n* = 30; TAU *n* = 18) showed a dropout rate of 16%. In comparison with TAU group, MFG group reported statistically significant gains in social functioning as indicated by the SNQ mean score (*p* = 0.002,|*d*| = 0.91), with increases in social contact frequency (*p* = 0.000, |*d*| = 1.08). Improvements in family coping skills were also notable, with statistically significant enhancements in the FCQ subscales for information (*p* = 0.003,|*d*| = 1.16) and avoidance reduction (*p* = 0.000,|*d*| = 0.92).

**TABLE 2:** Independent sample *t*‐test (two‐tailed) comparing the mean differences between the experimental (MFG) and control (TAU) caregivers groups at 6 months (T1).

		MFG caregivers (*n* = 30)	TAU caregivers (*n* = 18)		
		Mean *±* SD	Mean *±* SD	*p*	*|d|*
SNQ	Mean score	2.9 ± 0.4	2.6 ± 0.5	0.002**	**0.91** ^**††**^
	Social contact frequency	2.8 ± 0.5	2.3 ± 0.6	0.000***	**1.08** ^**††**^
	Practical support	3.2 ± 0.8	2.7 ± 0.8	0.018*	**0.65** ^**†**^
	Psychological support social network	3.1 ± 0.5	2.7 ± 0.7	0.019*	**0.64** ^**†**^
	Support from close individuals	3.1 ± 1.3	3.2 ± 1.3	0.433	0.50
FPQ	Help received	2.9 ± 0.5	2.6 ± 0.5	0.025*	**0.61** ^**†**^
	Objective burden	1.7 ± 0.6	1.7 ± 0.7	0.366	0.10
	Subjective burden	2.0 ± 0.6	1.7 ± 0.6	0.094	0.41
	Positive attitudes	2.7 ± 0.6	2.6 ± 0.5	0.264	0.19
	Critical or overprotective attitudes	1.8 ± 0.7	2.0 ± 0.7	0.188	0.27
FCQ	Information	3.3 ± 0.7	2.2 ± 1.1	0.003**	**1.16** ^**††**^
	Communication	3.5 ± 0.5	3.5 ± 0.3	0.422	0.06
	Social interests	3.2 ± 0.6	2.8 ± 0.6	0.015*	**0.71** ^**†**^
	Coercion	1.8 ± 0.6	1.7 ± 0.7	0.282	0.18
	Avoidance	1.7 ± 0.9	1.1 ± 0.2	0.000***	**0.92** ^**††**^
ABC	Total score	86.5 ± 18.1	72.7 ± 23.1	0.015*	**0.69** ^**†**^
GSQ	Mean score	1.9 ± 0.5	2.2 ± 0.8	0.066	0.49

Abbreviations: ABC, Italian ABC Questionnaire; FCQ, Family Coping Questionnaire; FPQ, Family Problems Questionnaire; GSQ, General Satisfaction Questionnaire 20.2; SNQ, Social Network Questionnaire.

Bold values marked with *, **, and *** indicate significant comparisons, which are denoted as 0.05, 0.01, and 0.001, respectively.

Bold values marked with **†** indicate moderate effect size (|*d*| > 0.6) and **††** very large effect size (|*d*| > 0.9).

### 3.6 Linear Mixed Model Analysis

The results revealed a significant Group × Time interaction for several outcome measures, indicating that caregivers in the MFG group improved significantly more over time compared to those in the TAU group. Specifically, in the SNQ, the Psychological Support subscale showed a significant interaction effect (*F*(1, 47) = 4.216, *p* = 0.046). Within the FPQ, significant Group × Time interactions were observed for Help Received (*F*(1, 47) = 7.596, *p* = 0.008) and for Critical or Overprotective Attitudes (*F*(1, 46) = 5.257, *p* = 0.026). The FCQ also showed significant interaction effects, particularly in the Communication subscale (*F*(1, 50) = 6.673, *p* = 0.013) and in the Coercion subscale (*F*(1, 43) = 14.121, *p* = 0.001). Finally, analysis of the GSQ revealed a significant Group × Time interaction for the total mean score (*F*(1, 45) = 5.557, *p* = 0.023).

## 4 Discussion

This study aimed to evaluate the impact of the MFG intervention on caregivers of individuals with BPD. The findings of this study suggest that participation in the MFG intervention was associated with significant improvements in caregiver outcomes compared to TAU. Caregivers in the MFG group reported enhanced social functioning, greater perceived psychological and practical support, and improved coping strategies within the family context. These improvements were evident across multiple domains, including communication, information sharing, and reduced use of coercive or avoidant strategies. Importantly, the longitudinal analysis confirmed that these positive changes were significantly greater over time in the intervention group compared to controls, highlighting the potential effectiveness of the MFG model in supporting families of individuals with BPD in real‐world clinical settings. Given the small sample size, these findings should be interpreted as exploratory and hypothesis generating, warranting replication in larger and more powered studies.

At baseline, our findings indicate that family members caring for individuals with BPD experience levels of burden comparable to those reported in caregivers of individuals with schizophrenia, a population more extensively studied in the context of family burden. Although our data were stored in aggregate form, limiting the ability to analyze item‐level responses, the total scores for objective (1.9) and subjective burden (2.1) indicate a moderate level of impact on both the emotional and practical aspects of caregivers' lives. This similarity is supported by earlier research showing that objective and subjective burden scores in schizophrenia caregivers range from 1.4 to 1.9 and from 1.6 to 2.3, respectively, across various European cities (Magliano et al. 1998). Our findings are consistent with Scheirs and Bok (2007), who observed that BPD caregivers report burden levels comparable to those in schizophrenia (Scheirs and Bok 2007). However, the underlying nature of caregiver burden may differ substantially between these two conditions. Schizophrenia is typically associated with pervasive impairments in personal autonomy and self‐determination, often requiring continuous support from family members (Kuipers 1993; Jones 1996; Awad and Voruganti 2008; Hayes et al. 2015). These caregiving demands can result in chronic stress, reduced social participation, and financial strain. In contrast, individuals with BPD may retain functional independence in areas such as employment or living arrangements, but their caregivers often face emotional and relational instability, including impulsive behavior, acute conflict, and limited emotional reciprocity (Gunderson et al. 1997; Ekdahl et al. 2011). One commonality across both groups is the often reported perception of insufficient empathy, support, and understanding from mental health services (Giffin 2008; Ekdahl et al. 2011). Our findings reinforce previous calls for improved education, communication, and collaboration between clinicians and family members (Stengård et al. 2000; Dunne and Rogers 2013; Lawn and McMahon 2015).

Although individuals with BPD have long received clinical attention, significant progress in developing structured evidence‐based treatments has occurred only in recent decades (Gunderson et al. 2018; Rossi and Ridolfi 2021). Historically, caregivers of people with BPD have often been blamed for the patient's condition and excluded from treatment processes, reflecting outdated stigmatizing theories such as the concept of the schizophrenogenic mother (Hartwell 1996). However, their role has evolved into that of essential collaborators, now recognized as crucial co‐therapists on the path to recovery (Santisteban et al. 2003; Porr 2010). Today, international and national guidelines emphasize the importance of involving family members and caregivers in treatment programs for BPD (NICE 2009; NHMRC 2012). In Emilia‐Romagna, the Regional Guidelines on Serious Personality Disorders support integrated and multidisciplinary approaches, explicitly encouraging the inclusion of family members in psychoeducational and therapeutic interventions (Regione Emilia Romagna 2024). These guidelines are in line with global best practices, and, compared to other Italian regions, Emilia‐Romagna has been proactive in implementing structured programs that adhere to these recommendations, promoting collaboration between mental health services and family members.

In line with the evolving recognition of caregivers' critical roles in the treatment of BPD, interventions for family members have significantly developed over recent decades to address their growing importance in supporting individuals with this disorder. The MFG therapy focuses on the caregiver's social functioning and coping (Gunderson et al. 1997). Consistent with findings from Gunderson's 1997 pilot study, our research demonstrated significant improvements in outcome measures related to social functioning and coping skills among family members (Gunderson et al. 1997). Additionally, our study highlighted high rates of treatment completion, cost‐effectiveness, and good feasibility within CMHCs offering dedicated BPD programs. Other existing treatments for families of individuals with BPD include Dialectical Behavior Therapy—Family Skills Training (DBT‐FST), which was among the first short‐term interventions focused on emotional regulation and creating a healthier family environment (Hoffman et al. 1999). Similarly, Family Connections (FC) (Hoffman et al. 2005), a psychoeducational model, has shown consistent effectiveness in improving emotional regulation, family functioning, and caregiver burden. It has been praised for its accessibility and affordability, with benefits maintained at follow‐up (Flynn et al. 2017; Liljedahl et al. 2019; Courey et al. 2021; Guillén 2024). Making Sense of BPD (MS‐BPD) and Mentalization‐Based Family Intervention (MBT‐FACTS) have also demonstrated efficacy in improving family knowledge, communication, and overall functioning (Pearce et al. 2017; Bateman and Fonagy 2019; Betts et al. 2023). More recently, new online interventions, such as moderated online social therapy for family and friends of young people with BPD, are emerging (Gleeson et al. 2021), offering promising accessibility benefits despite some initial resistance from clinical staff toward the adoption of digital formats (Orsolini et al. 2022; Mendes‐Santos et al. 2022).

Our study on MFG provides evidence for the effectiveness of a structured yet accessible intervention that can be implemented within generalist CMHCs. One of the key strengths of the model is its feasibility: it can be delivered by clinicians without requiring extensive or specialized training, making it well suited for real‐world settings where resources may be limited. The relatively short duration of the intervention further supports its scalability and accessibility for a wide range of families. Our findings suggest that this approach can meaningfully enhance family functioning, with particular improvements observed in areas such as social engagement and reduced avoidant behaviors. These changes are especially relevant given the social and emotional challenges that often characterize the caregiving experience in BPD.

This study exhibits limitations that should be considered. The main limitation was the overall small sample size, which, along with resource constraints, may have reduced the statistical power to detect more subtle effects and limited the generalizability of the findings. Second, our power analysis assumed a large effect size in the absence of prior data on this specific MFG intervention; while this is a common challenge in early‐stage research, it introduces uncertainty and should be interpreted with caution. Third, the use of block randomization, combined with the exclusion of participants with incomplete baseline assessments, resulted in an uneven allocation of participants between groups. While this did not compromise the integrity of the randomization process, it may have introduced minor imbalances that should be considered when interpreting the results. Fourth, while previous studies on FC and MS‐BPD have shown efficacy in reducing family subjective and objective burden (Rajalin et al. 2009; Cohen et al. 2024), in our study, no significant changes were observed in either variable as measured by the FPQ. This could be attributed to our small sample size or the possibility that both the MFG and TAU groups experienced improvements in burden, limiting the ability to detect between‐group differences. Fifth, the psychopathological status of family members was not assessed, which may have influenced their reported experiences of burden and coping. Finally, the tools used to measure caregiver burden were originally designed for schizophrenia, and while they provided useful insights, they may not fully capture the specific challenges associated with BPD caregiving (Magliano et al. 2000; Magliano et al. 2005). Furthermore, all responses were stored in aggregate form as total scores rather than individual item responses. As a result, it was not possible to calculate internal consistency measures (e.g., Cronbach's alpha) for our sample.

In conclusion, this study highlights the potential of MFG therapy as a valuable intervention for caregivers of individuals with BPD. Findings suggest that MFG therapy can significantly improve social functioning and coping skills among family members, equipping them with better tools to face the challenges associated with caregiving. We believe that offering psychoeducational interventions to caregivers at an early stage can be beneficial in terms of reducing their suffering and improving distressed family dynamics. Given its feasibility, effectiveness, and alignment with the needs of real‐world services, the MFG intervention shows potential for integration into standard CMHC protocols and caregiver‐focused service pathways, contributing to family‐inclusive models of care for individuals with BPD.

## Author Contributions

Conceptualization: M.M., D.B., B.B., and F.M. Methodology: S.T.V. and B.B. Formal analysis: S.T.V. and F.M. Investigation: B.B., F.M., and P.T. Data curation: P.T. and S.T.V. Writing—original draft preparation: S.T.V. and F.M. Writing—review and editing: B.B. and M.M. Supervision: A.R.A., D.B., and D.D.R. All the authors have read and approved the final manuscript.

## Ethics Statement

The present research was conducted in alignment with the regional guidelines established in the “Dossier No. 240/2014—Regional Program for EDs—Contributions 2009–2012.” This study was approved by the Local Ethics Committee (Prot. No. 360–2022‐OSS‐AULSBO 05‐18‐2022) and conducted in accordance with the ethical principles outlined in the Declaration of Helsinki and Good Clinical Practice guidelines.

## Conflicts of Interest

The authors declare no conflicts of interest.

## Consent

All participants involved in this study provided informed consent prior to their participation. Both patients and their caregivers were asked to provide informed consent to participate in this study. While dual consent was preferred, cases in which only the caregiver provided consent were still included, as this study aimed to assess caregivers' psychometrics independently of patient participation. Although this situation did not occur in practice, this study design allowed for the inclusion of caregivers in such cases.

## Permission to Reproduce Material From Other Sources

Permission is granted to reproduce any material from this work, provided that appropriate acknowledgment of the source is made and that the reproduction complies with applicable copyright laws and ethical standards.

## Data Availability

The data that support the findings of this study are available upon request from the corresponding author. The data are not publicly available because of privacy or ethical restrictions.

## Appendix

**Table:**

Phase	Content
Phase 1: Family training	The first phase focuses on providing families with knowledge about BPD through psychoeducation. Information regards the core symptoms of BPD, etiology, the impact of emotional dysregulation, impulsivity, and instability on the patient's behavior. Psychoeducation is complemented by discussions with the objective to normalize family experiences and promoting mutual understanding in a non‐stigmatizing environment. Strategies include clarifying misconceptions about BPD and teaching family members to recognize maladaptive patterns, such as invalidation or critical attitudes, that may unintentionally exacerbate the patient's symptoms.
Phase 2: Skills acquisition	The second phase involves teaching practical skills to manage common challenges associated with BPD. The main focus is on communication improvement, anger management, and strategies to manage self‐injurious behaviors. Families are trained to use validation techniques to de‐escalate emotional conflicts and establish a non‐confrontational environment. Communication exercises focus on expression of needs and active listening. Anger management sessions teach strategies such as time‐outs, relaxation techniques, and reframing to diffuse tension during intense emotional interactions. For self‐injurious behavior management, family members learn to identify warning signs and respond without reinforcing distress.
Phase 3: Consolidation of changes	The third phase's aim is to consolidate the skills learned. The therapist takes a less directive role and promotes active problem solving within the group. Role‐playing exercises are used to simulate challenging scenarios, such as managing emotional outbursts or conflict resolution, allowing families to practice new skills in a supportive environment. A peer support is encouraged within the group by fostering a sense of community and shared learning. At the end of this phase, families should demonstrate greater independence in managing interpersonal challenges and maintaining a stable and empathetic relationship with the patient.
